# AQP9-induced cell cycle arrest is associated with RAS activation and improves chemotherapy treatment efficacy in colorectal cancer

**DOI:** 10.1038/cddis.2017.282

**Published:** 2017-06-22

**Authors:** Dandan Huang, Xingzhi Feng, Yiting Liu, Yanhong Deng, Hao Chen, Daici Chen, Lekun Fang, Yue Cai, Huanliang Liu, Lei Wang, Jianping Wang, Zihuan Yang

**Affiliations:** 1 Guangdong Provincial Key Laboratory of Colorectal and Pelvic Floor Diseases, The Sixth Affiliated Hospital, Sun Yat-sen University, Guangzhou, Guangdong 510655, China; 2 Guangdong Institute of Gastroenterology, Guangzhou, Guangdong 510655, China; 3 Department of Medical Oncology, The Sixth Affiliated Hospital, Sun Yat-sen University, Guangzhou, Guangdong 510655, China

## Abstract

Aquaporin-9 (AQP9) expression is associated with arsenic sensitivity in leukemia cells. However, the role of AQP9 in regulating tumor sensitivity to adjuvant chemotherapy in colorectal cancer (CRC) has not been elucidated. In this study, we demonstrated that AQP9 can serve as an independent predictive marker for adjuvant chemotherapy in CRC. Patients with high AQP9 expression had higher rate of disease-free survival (DFS) than those with low AQP9 expression. Upregulation of AQP9 was associated with enhanced chemosensitivity to 5-fluorouracil (5-FU) both *in vitro* and *in vivo*. Overexpression of AQP9 resulted in an increased intracellular level of 5-FU in CRC cells, hence leading to a higher percentage of apoptosis after 5-FU treatment. Moreover, AQP9 is positively associated with RAS activation and other downstream signaling molecules in CRC. AQP9 overexpression resulted in p21 upregulation and induced S-phase arrest. Taken together, AQP9 enhances the cytotoxic response to 5-FU in CRC cells by simultaneously inducing S-phase arrest via activation of RAS signaling and facilitating drug uptake. Our results suggest that AQP9 might be a novel predictor for the benefit of 5-FU-based chemotherapy in CRC. The identification of AQP9-induced tumor sensitivity to 5-FU highlights the role of AQP9 in regulating chemosensitivity in CRC.

CRC incidence in China is growing at the rate of 3.9%. There were about 376 000 new cases in 2015.^1^ 5-FU-based chemotherapy regimens are routinely employed to treat patients at high risk of developing recurrence or those with metastatic disease. However, up to 40% of advanced CRC patients receiving chemotherapy do not derive benefit from the treatment and will eventually experience recurrence.^2^

Several clinical studies have been attempted to find markers capable of predicting the benefit from chemotherapy. However, few of the markers have been translated into clinical management of CRC. The strongest candidate so far appears to be microsatellite instability (MSI).^3^ Previous studies demonstrated that 5-FU-based chemotherapy was specifically harmful for microsatellite-unstable (MSI-H) stage II disease.^4, 5^ Meanwhile, other studies suggested that there was no differential response related to MSI status.^6, 7^ The role of MSI in stage III CRC is even more controversial.^8, 9^ Therefore, defining new predictive markers for patients to derive clinical benefit remains a major challenge in current CRC therapies.

Our previous microarray analysis showed that AQP9 was upregulated in stage III CRC adjuvant chemotherapy responders.^10^ However, the mechanism underlying the effects of AQP9 on the regulation of chemosensitivity in CRC is unclear. AQP9 belongs to the aquaglyceroporin subfamily of aquaporins and is permeable to water and small neutral molecules. Modulation of aquaporins’ function or expression could have therapeutic potential in many diseases, including cancer.^11, 12^ Studies have shown that AQP3, another aquaglyceroporin, was involved in cell proliferation and may be attributed to tumor initiation.^13, 14, 15^ Evidence showed that AQP9 has a vital role in modulating arsenite sensitivity in leukemia.^16, 17^ The role of AQP9 in regulating chemosensitivity in CRC warrants further investigation.

In the current study, we aimed to explore the association between AQP9 expression and FOLFOX-based adjuvant chemotherapy outcome in CRC patients. Our results demonstrated for the first time that AQP9 expression level is correlated with adjuvant chemotherapy response in CRC, particularly in stage III CRC patients.

## Results

### AQP9 expression is positively correlated with better disease-free survival (DFS) in CRC patients treated with chemotherapy

To investigate whether AQP9 expression was associated with chemosensitivity in CRC, we first compared AQP9 mRNA levels in CRC tumor tissues from 16 chemotherapy responders and 16 nonresponders. Tumors in responders showed much higher expression level of AQP9 compared with nonresponders (*P*=0.014; Supplementary Figure S1a).

To further determine the clinical relevance, we analyzed the AQP9 expression in tumor tissue microarray from 367 stage II and III CRC patients by immunohistochemical (IHC) staining (Supplementary Figure S1c). Among these patients, 234 received 5-FU-based chemotherapy. Patients not receiving adjuvant chemotherapy were more likely to have stage II disease and tended to be older (Table 1). AQP9 low expression was significantly associated with poor tumor differentiation (*P*=0.004) (Table 1). AQP9 expression level was significantly higher in colon compared with that in rectum (*P*=0.037). No other significance was observed. Additionally, we analyzed gene expression data from The Cancer Genome Atlas (TCGA) database. By analyzing the available TCGA RNA-Seq data with clinical information downloaded on 16 December 2015 (*n*=189), we found that AQP9 expression is positively correlated to primary tumor pathological spread in CRC (Supplementary Figure S1b).

**Figure 1:**
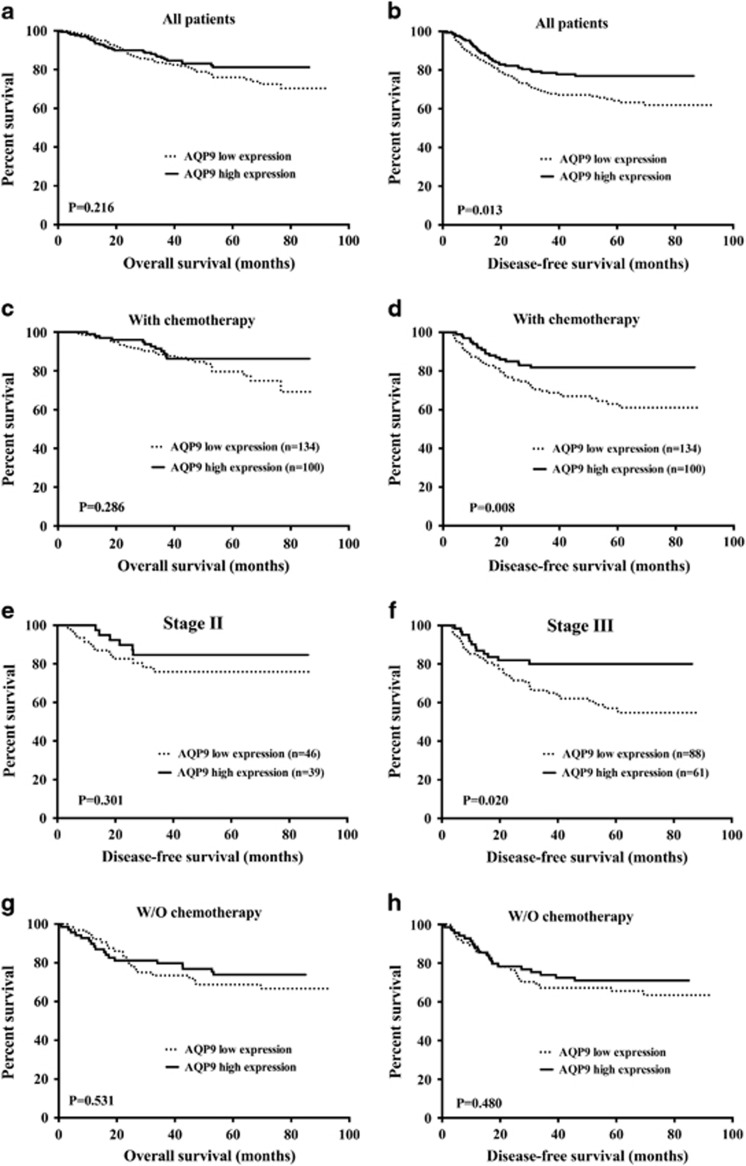
Correlation between expression of AQP9 and chemotherapy treatment outcome. Kaplan–Meier survival curves of (**a**) OS and (**b**) DFS in 367 CRC patients by AQP9 status. (*P*=0.216 and *P*=0.013, respectively). (**c**) OS and (**d**) DFS in 234 CRC patients with chemotherapy by AQP9 status (*P*=0.286 and *P*=0.008, respectively). Increased level of AQP9 is significantly correlated with longer DFS in CRC patients. (**e**) DFS in patients with chemotherapy in stage II disease by AQP9 status (*P*=0.301). (**f**) DFS in patients with chemotherapy in stage III disease by AQP9 status (*P*=0.020). (**g**) OS and (**h**) DFS in 133 CRC patients without chemotherapy by AQP9 status (*P*=0.531 and *P*=0.480, respectively). The log-rank analysis was used to test for significance

Kaplan–Meier analysis showed that high AQP9 expression was associated with improved DFS (Figure 1b; *P*=0.013), especially in those who received adjuvant chemotherapy (Figure 1d; *P*=0.008). Further analysis indicated that this benefit was mainly attributed to the significant improvement of DFS in stage III and AQP9 high expression tumors in chemotherapy subgroups (Figures 1e and f). However, the analysis of patients who did not receive adjuvant therapy failed to show any significant differences in overall survival (OS) and DFS by the AQP9 expression level (Figures 1g and h). Multivariate Cox regression analysis for all 367 patients showed that pN and treatment status are independent factors for both OS and DFS (Table 2). Although there is no significant association between AQP9 status and OS, the AQP9 high expression group tends to have a better DFS (Table 2; *P*=0.061). By multivariate analysis of 234 patients with chemotherapy adjusted for age, stage, grade and site, we found that the AQP9 expression level was significantly associated with DFS (Supplementary Table S1, *P*=0.023) but not with OS (Supplementary Table S2). Therefore, AQP9 might be a novel marker that can predict chemotherapy treatment outcomes of CRC patients, especially for stage III disease.

### AQP9 expression is positively correlated with CRC cell sensitivity to 5-FU

**Figure 2:**
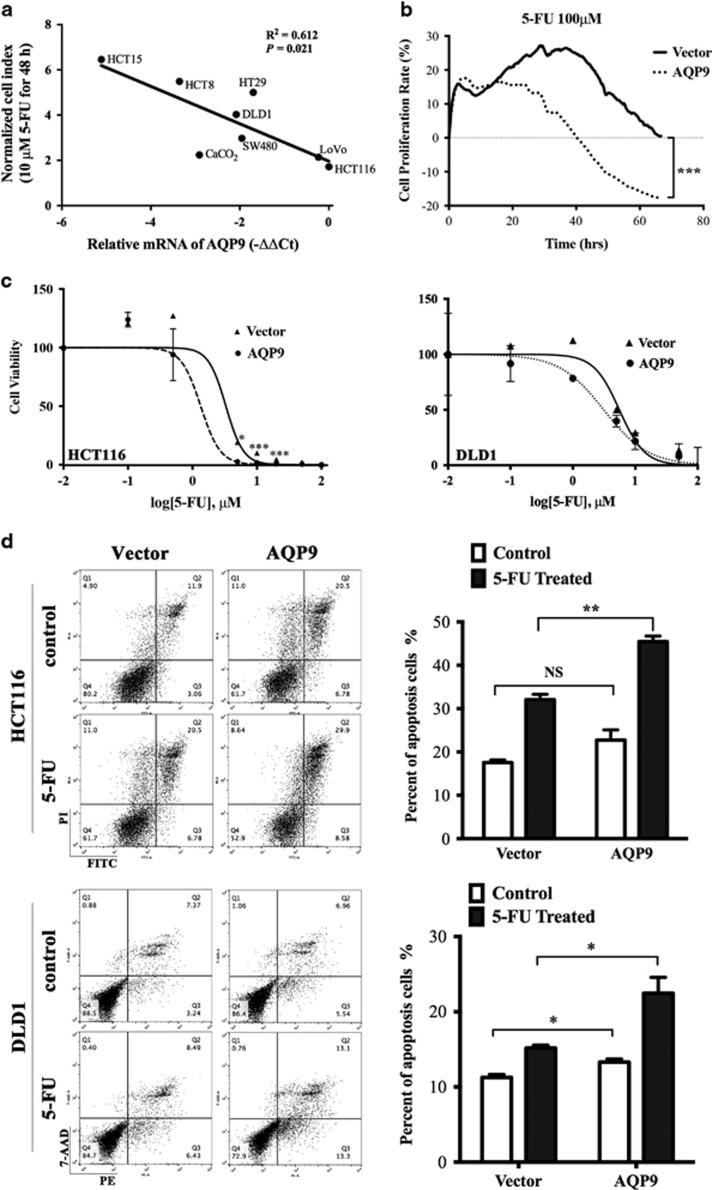
AQP9 overexpression increased chemosensitivity in CRC cells. (**a**) AQP9 mRNA quantity determined by quantitative PCR was inversely related to cell proliferation index after treatment in 10 *μ*M 5-FU for 48 h. In all, 10^4^ CRC cells were seeded in E-Plates and cell growth was continuously monitored in real-time using an xCELLigence RTCA DP instrument. Each point represents the mean±S.E.M. of four different experiments. (**b**) Cell index of AQP9-overexpressed HCT116 cells dropped more rapidly than control cells upon 5-FU treatment. (**c**) HCT116 and DLD1 cells were treated with increasing concentrations of 5-FU from 0 to 200 *μ*M after transfection with AQP9 or control. AQP9 overexpression gives rise to higher sensitivity to 5-FU as indicated by IC50. (**d**) Flow cytometer analysis indicated that 5-FU significantly increased the portion of apoptotic cells in AQP9-overexpressed cells. HCT116 and DLD1 cells transfected with AQP9 or control vectors were treated with 100 *μ*M 5-FU for 24 h. Results are the means±S.E.M. of three independent experiments, each performed in duplicates. NS, not significant; *P<0.05; **P<0.01; ***P<0.001 by the two-tailed Student’s *t*-test

To further investigate whether AQP9 level was associated with CRC cell sensitivity to 5-FU *in vitro*, 8 CRC cell lines were treated with 10 *μ*M 5-FU for 48 h. AQP9 level determined by qPCR was negatively correlated with cell proliferation index measured at the end of treatment (*P*=0.021; Figure 2a), indicating that the higher the AQP9 level, the more the cells were killed by 5-FU. Indeed, in the presence of 5-FU, cell growth curve started to drop earlier in AQP9-overexpressed cells than in control cells (Figure 2b). Cells overexpressing AQP9 showed enhancement of cytotoxicity across a range of concentrations of 5-FU after 48 h of exposure (Figure 2c). We then evaluated the influence of AQP9 on 5-FU-induced apoptosis in HCT116 and DLD1 cells by flow cytometry. Apoptotic responses measured after 24 h of 5-FU exposure were substantially enhanced in AQP9 cells (Figure 2d). Taken together, these results suggest that AQP9 overexpression increases cell sensitivity to 5-FU.

### AQP9 facilitates 5-FU uptake into CRC cells

**Figure 3:**
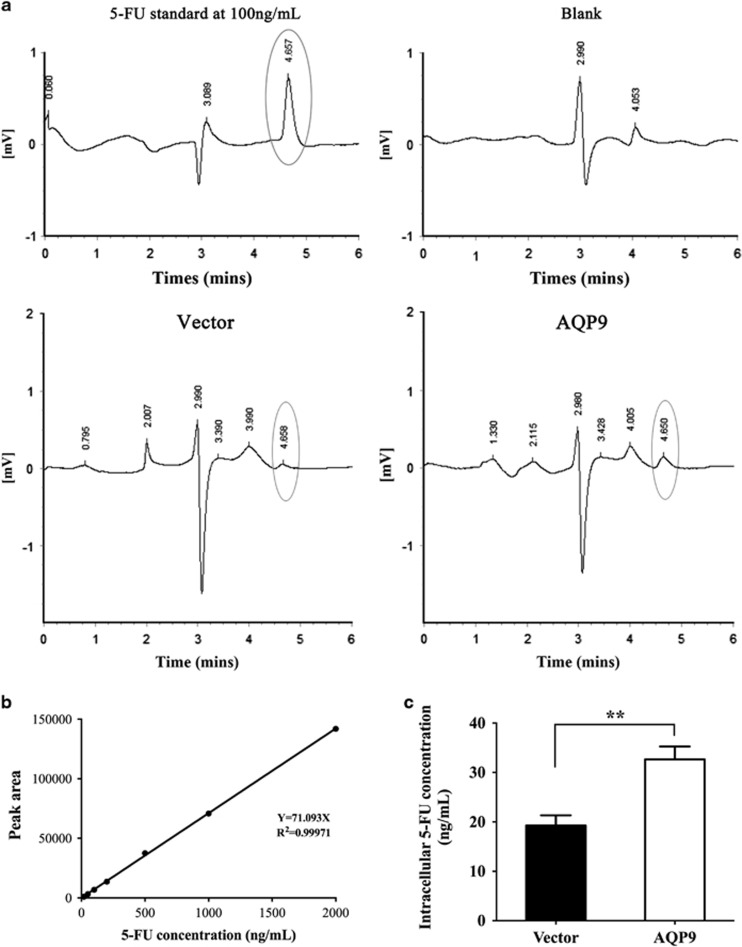
AQP9 facilitates 5-FU uptake in CRC cells. (**a**) Representative chromatogram of 5-FU detected in lysates of HCT116 cells. (**b**) Standard curve using peak area against standard 5-FU at different concentrations. (**c**) Concentrations of 5-FU detected in lysates of HCT116 cells transfected with control vector and AQP9. Intracellular 5-FU levels following a 24 h incubation with 5-FU (50 *μ*g/ml) are shown as ng/ml. **P<0.01 by the two-tailed Student’s *t*-test. Results were replicated (*n*⩾5 experiments)

Given that AQP9 can transport glycerol and other small molecules,^18^ we hypothesized that AQP9 could enhance chemosensitivity by facilitating 5-FU uptake into CRC cells. To confirm the role of AQP9 in 5-FU entry and sensitivity, we used a reverse-phase, high-performance liquid chromatography (HPLC) system to measure the intracellular 5-FU concentration in CRC cells. Following 24 h incubation with 50 μg/ml 5-FU, we observed that the intracellular 5-FU levels are approximately two fold higher in the lysates of cells transfected with AQP9 compared with vectors (Figure 3, *P*=0.003), suggesting that the overexpression of AQP9 enhances 5-FU uptake into CRC cells.

**Figure 4:**
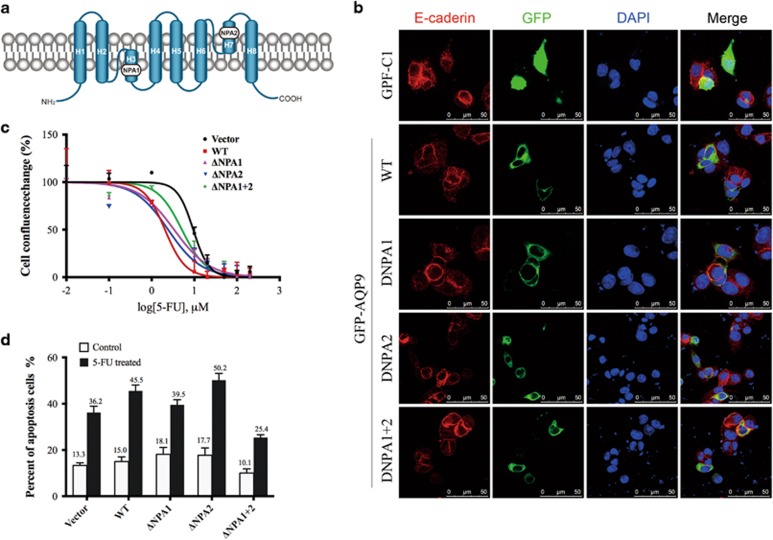
Effect of NPA deletion of AQP9 on cell sensitivity to 5-FU. (**a**) Schematic illustration of AQP9 transmembrane domains and NPA motifs. (**b**) AQP9 localization was determined by GFP immunofluorescence (green) using confocal microscope. Cells were stained with an antibody against E-cadherin (red) for membrane localization. The nuclei were counterstained with DAPI (blue). GFP was distributed throughout the whole cell in cells transfected with empty vector; however, it was clearly located on the membrane in cells transfected with WT or NPA mutants. (**c**) Effect of NPA mutation on chemosensitivity to 5-FU was determined by cell confluence. Cells treated with various concentrations of 5-FU were monitored for 72 h using the IncuCyte ZOOM instrument. (**d**) The percentage of apoptosis induced by 5-FU was determined in HCT116 cells transfected with AQP9-WT plasmid or NPA-mutated plasmids or an empty vector. After treatment with 5-FU (100 *μ*M, 24 h), apoptosis was assessed by FACS analysis

The two NPA (Asn-Pro-Ala) signature motifs (Figure 4a) conserved throughout the AQP superfamily are important for selective pore formation and transportation function of AQP9.^19, 20^ To gain insight into NPA motifs of AQP9 in regulating chemosensitivity to 5-FU, we generated three AQP9 mutants with NPA1 deletion, NPA2 deletion and NPA1+2 double deletion, respectively. HCT116 cells were transfected with AQP9-WT plasmids and NPA mutants. Immunofluorescence analysis indicated that WT-AQP9 as well as AQP9 mutants were expressed and localized on the plasma membrane, indicating that the NPA deletion does not alter cellular localization pattern of AQP9 (Figure 4b). NPA mutants showed increased resistance to 5-FU than WT-AQP9 cells (Figure 4c). In flow cytometric analysis, 5-FU-induced apoptotic cell percentage was much lower in NPA1 and NPA1+2 deletion cells than that in WT-AQP9 cells (Figure 4d).

### AQP9 overexpression induces S-phase arrest and RAS signaling activation

**Figure 5:**
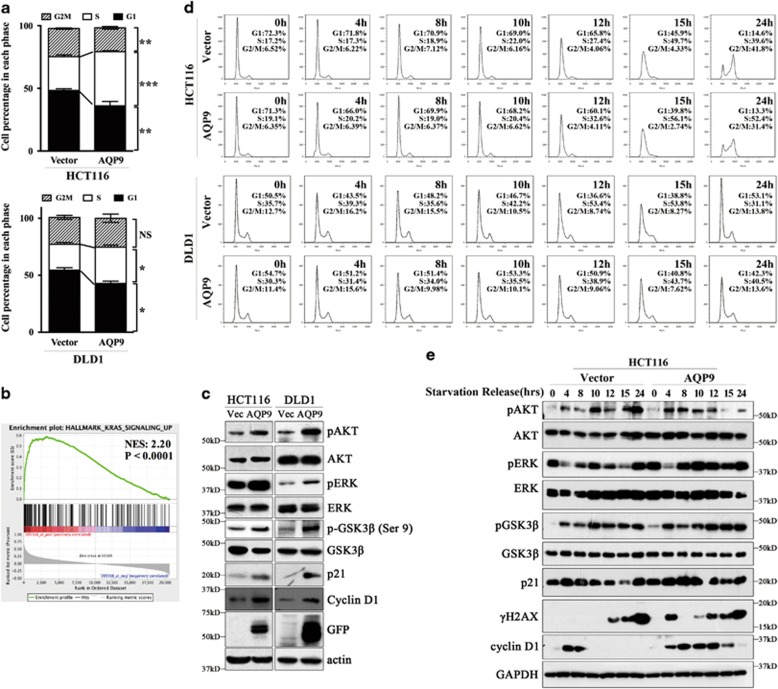
AQP9 regulates CRC cell cycle progression through activation of RAS signaling pathway. (**a**) Cell cycle analyses determined by FACS. Results showed that AQP9 overexpression increases the percentage of cells in S phase. (**b**) Gene set enrichment analysis was performed in GSE14333. AQP9 overexpression positively correlates with genes involved in KRAS activation. (**c**) AQP9-overexpressing cells displayed increased activation of AKT, ERK and P21. (**d**) HCT116 and DLD1 cells transfected with control vector and AQP9 were synchronized by serum starvation for 48 h and induced to re-enter the cell cycle by the addition of serum over 0–24 h. Cells were harvested for PI staining and analyzed by FACS to determine the cell cycle fraction. FACS plots and data are representative of at least three separate experiments. (**e**) Activation of Ras signaling, p21, H2AX and GAPDH proteins were analyzed by western blotting analysis. NS, not significant; *P<0.05; **P<0.01; ***P<0.001 by the two-tailed Student’s *t*-test

Because 5-FU exerts its anticancer effect through incorporation of FdUTP into DNA and RNA specifically during S phase,^21^ cancer cells in the S phase are more susceptible to 5-FU-induced cell death.^22^ We therefore sought to determine whether AQP9 overexpression has an effect on cell cycle regulation and, more specifically, whether it increases the toxicity of 5-FU. HCT116 and DLD1, two CRC cell lines, were transfected with control or AQP9 plasmid. We observed an increase in the percentage of cells in the S phase for AQP9-overpressing cells (Figure 5a). To understand the molecular mechanism whereby AQP9 leads to S-phase arrest in CRC cells, we performed Gene Set Enrichment Analysis to investigate the critical signaling pathway associated with AQP9 overexpression in CRC (GEO: GSE14333, *n*=290). We found that gene sets of KRAS signaling pathway were enriched in samples with high expression level of AQP9 (Figure 5b). RAS is frequently mutated in CRC and activates several downstream effectors.^23^ The most intensive studied pathways are RAF/MAPK and PI3K/AKT,^23^ which are often concurrently activated in many cancers and inhibiting GSK3 by phosphorylating the same residue.^24, 25^ Immunoblot analysis showed that phosphorylation of AKT, ERK and GSK3*β* were increased in AQP9-overexpressing CRC cells (Figure 5c). Furthermore, AQP9 overexpression also induced an increase in the levels of p21. Increase of GSK-3 activity can trigger proteasomal degradation of p21, whereas the inhibitory phosphorylation of GSK3*β* (Ser 9) results in an increase in p21 levels.^26^ Our findings indicate that AQP9 overexpression is associated with RAS activation, which may subsequently inhibit GSK3*β* activity and increase p21 level. As p21 is crucial for cell cycle regulation and cellular differentiation,^27, 28^ we performed cell cycle synchronization experiments to further elucidate the role of AQP9 in modulating cell cycle. Control and AQP9-overexpressing cells were synchronized by serum starvation for 48 h and then released by addition of serum over 0–24 h. The cell cycle distribution was determined by PI staining. Serum deprivation resulted in G0/G1 arrests in both control and AQP9-overexpressing cells. After serum addition, both cells re-entered the cell cycle with increase of S-phase fraction. Cell cycle distribution was similar in control and AQP9-overexpressing cells at 4, 8 and 10 h. While with the time release from serum addition from 18 to 24 h, we observed that AQP9-overexpressing HCT116 cells showed significantly higher S-phase fraction than the control cells (Figure 5d and Supplementary Figure S2b). The results indicated that AQP9-overexpressing CRC cells have a retarded cell cycle progression from S phase to G2/M phase, compared with control cells. Western blotting analysis showed that AQP9 overexpression led to an apparent upregulation of pAKT, pERK, pGSK3*β* and p21 starting at 4 h after serum re-addition (Figure 5e). Noticeably, the *γ*H2AX level started to increase earlier in AQP9-overexpressing cells than in control cells. Moreover, we observed an elevated nuclear accumulation of *γ*H2AX in AQP9-overexpressing cells (Supplementary Figure S2a). Enhanced phosphorylation of histone H2AX (*γ*H2AX) has been shown to occur in response to hypertonic condition and DNA damage via the DNA-PK/GSK3*β* pathway.^29, 30^ H2AX is also required for increasing p21 levels and subsequently results in checkpoint activation and cell cycle arrest.^31, 32^ Hence, we speculated that the retarded cell cycle progression in cells overexpressing AQP9 might be due to more time needed for DNA repair in these cells. Similar results were also obtained in DLD1 cells (Figure 5d and Supplementary Figure S2c).

### Mice overexpressing AQP9 are more sensitive to 5-FU chemotherapy

**Figure 6:**
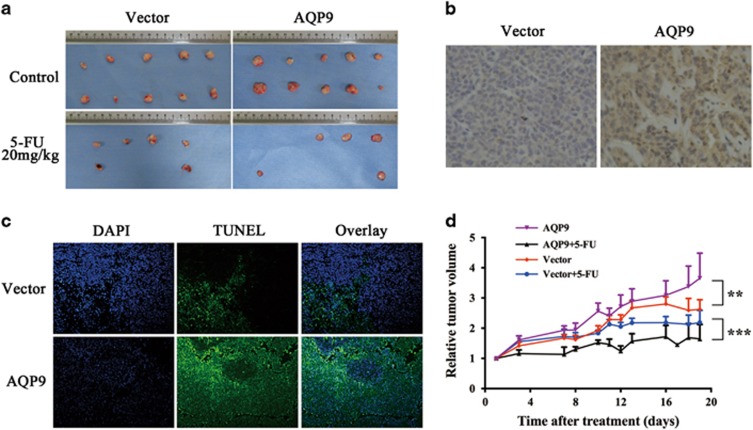
Effect of AQP9 on CRC cell line xenografts and 5-FU sensitivity in nude mice. (**a**) Images of tumors generated in different groups of mice after intraperitoneal bolus treatment with 20 mg/kg 5-FU or equal volume of PBS as control. Some of the tumors completely disappeared after 5-FU treatment. (**b**) IHC staining of AQP9 in tumor tissues from control vector and AQP9 group. (**c**) Cell death induced by 5-FU treatment was assessed by DNA strand breakage using a TUNEL (terminal deoxinucleotidyl transferase-mediated dUTP-fluorescein nick end labeling) analysis. Cell nuclei were detected as blue and cells with DNA strand breakage as green. (**d**) Normalized tumor growth curves (relative tumor volume) after treatment. Data are shown as the mean±S.E.M. of 10 mice for each group. **P<0.01; ***P<0.001 by two-way analysis of variance

To investigate the *in vivo* consequences of AQP9 expression in chemotherapy sensitivity, we implanted HCT116_AQP9_ or HCT116_vec_ cells into nude mice. In each group, half of the mice received phosphate-buffered saline (PBS) as control and half received 5-FU treatment. IHC analysis confirmed that the AQP9 expression level was higher in the HCT116_AQP9_ groups than in the HCT116_vec_ groups (Figure 6b). To determine 5-FU-induced cell death, we performed TUNEL analysis on tumor tissue resected from treated mice. The data demonstrated that significantly higher cell death was induced by 5-FU in the HCT116_AQP9_ groups than in the HCT116_vec_ groups (Figure 6c). 5-FU reduced tumor growth in both the treatment groups. Moreover, the antitumor effect of 5-FU was enhanced in the HCT116_AQP9_ group than in the HCT116_vec_ group (Figure 6d). These *in vivo* results are consistent with our *in vitro* observations that CRC cells expressing higher level of AQP9 showed increased sensitivity to 5-FU.

## Discussion

FOLFOX-based chemotherapy for CRC treatment has been increasingly used to improve patient survival. Predictive molecular markers identifying which patients may benefit from the treatment may greatly improve the efficacy. In the present study, we investigated the mechanism of AQP9 in modulating CRC chemosensitivity. Noticeably, we found that high AQP9 is a predictive marker for stage III CRC patients with adjuvant chemotherapy. Moreover, we found that AQP9 functions as a drug transporter and further sensitized tumor cells to chemotherapy drugs associated with RAS signaling activation.

We found that the therapeutic response of 5-FU was enhanced in tumors containing AQP9-overexpressing cells, in comparison to controls. More importantly, CRC patients treated with chemotherapy in the AQP9 high expression subgroup showed significant better DFS. We also found a strong clinical association between AQP9 level and tumor differentiation grade. Although the OS is not significantly different between the AQP9 low and high groups, the trend is slightly better in the AQP9 high expression group. This might be due to the differentiation grade, which is usually higher in tumors with high level of AQP9. Collectively, these observations suggest that AQP9 expression may be a useful predictive marker and can be used to stratify patients into a group that would benefit from FOLFOX-based chemotherapy treatment.

The increased sensitivity to chemotherapy induced by AQP9 overexpression could be explained by multiple mechanisms. AQP9 is known to be involved in the uptake of arsenic, thus modulating As_2_O_3_-induced cytotoxicity in leukemia and other cancers.^16^ However, whether AQP9 could facilitate 5-FU uptake in CRC cells remained unknown. Our HPLC analysis showed that AQP9-overexpressing cells have higher intracellular 5-FU levels, which reflected an increase in sensitivity to 5-FU in those cells. It has been noted that AQP9 has two highly conserved NPA motifs of the AQP family.^33, 34^ Several mutagenesis studies indicate that mutations near the NPA motifs alter aquaporin function,^35, 36, 37, 38^ suggesting that this conserved region has a crucial role in membrane translocation and neutral solute transportation of aquaporins. In this way, we tested the effect of NPA deletion on sensitivity of HCT116 cells to 5-FU. Our results showed that NPA motif deletion did not alter AQP9 membrane location but reduced the sensitivity to 5-FU in CRC cells. Surprisingly, AQP9 ΔNPA2 deletion increased the 5-FU sensitivity compared with wild type. A possible explanation is that these two NPA motifs may have different roles in the formation of pore structure. Our future studies will address whether the two NPA mutations change the channel selectivity and permeability to solutes.

Antitumor drugs directly interfere with DNA replication specifically targeting cells in a particular cell cycle phase.^39^ Therefore, the position of tumor cells in the cell cycle and the ability to undergo apoptosis in response to drug treatment together have an important role in the sensitivity of tumor cells to chemotherapy. We found that AQP9-overexpressing cells exhibited a S-phase arrest phenotype compared with control cells. Furthermore, CRC cells with AQP9 overexpression displayed enhanced activation of RAS and downstream PI3K/AKT and ERK signaling, which have been shown to regulate cell differentiation and cell cycle arrest via inhibition of GSK3*β* and upregulation of p21.^40, 41^ Substantial evidences have indicated that p21 can promote cell differentiation and cellular senescence.^42^ Hence, this could also explain our clinical finding that AQP9 level is associated with tumor differentiation grade (Table 1). Aquaglyceroporin is a primary route of glycerol uptake and serves as a metabolic gateways.^43^ The synthesis of triacylglycerols from the esterification of glycerol and free fatty acids provides an important source of neutral lipids that are subsequently used during fatty acid oxidation to produce ATP. Thus AQP9-dependent regulation of glycerol transport promotes ATP production and may provide a complementary mechanism for activation of RAS and downstream signal pathways in CRC.

A number of research groups have shown that glucose condition was related to chemosensitivity.^44, 45^ Interestingly, gene expression data set analysis from the online database BioGPS (http://biogps.org/) showed that glucose regulates AQP9 expression level depending on KRAS status in CRC cells (GEO: GSE31084). High glucose downregulates AQP9 mRNA level in KRAS-mutant CRC cells. This trend was opposite to CRC cells with wild-type KRAS phenotype (Supplementary Figure S3). These collective findings indicate that the relationship between glucose and chemosensitivity might be associated with not only tumor metabolism but also AQP9 level and consequent drug uptake efficiency in cancer cells. The downregulation of AQP9 under high glucose condition in KRAS-mutant CRC cells may be a negative feedback of Ras signaling activation. For these reasons, the combination of chemotherapy and agents for regulating AQP9 expression represents a promising strategy to improve chemotherapeutic efficacy. AQP9 is not only a transmembrane channel that could facilitate drug uptake but also a gateway closely associated with energy metabolism. Simultaneous detection of AQP9 level and KRAS status and selective application of glucose may be a choice for increasing AQP9 level. Nevertheless, the crosstalk between AQP9 and cell metabolism in the modulation of chemosensitivity is worthy of further investigation.

**Figure 7:**
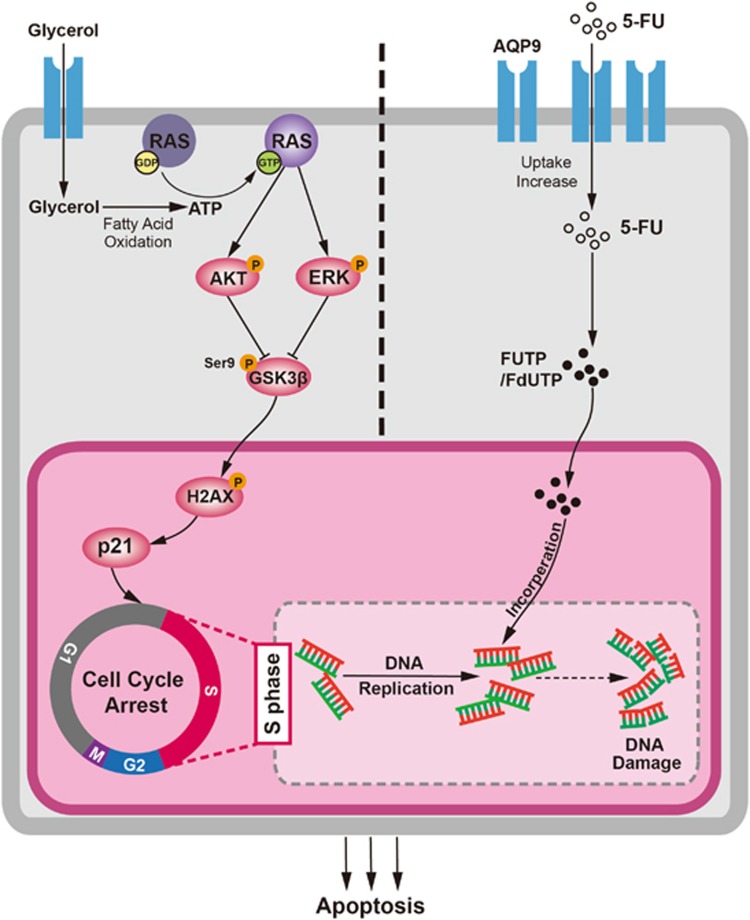
Proposed model of AQP9 in regulating 5-FU sensitivity in CRC. Glycerol uptake through AQP9 upregulation was increased. The synthesis of triacylglycerol from glycerol and free fatty acids provides an important source of neutral lipids that are subsequently used during fatty acid oxidation to produce ATP, thereby activating Ras and downstream pathways. Ras signaling activation then subsequently inhibit GSK3*β* activity and increase p21 level, which induces S-phase cell cycle arrest. On the other hand, AQP9 facilitates transmembrane uptake of 5-FU, hence sensitizes the cancer cells to 5-FU-mediated cytotoxicity.

In summary, for the first time we demonstrated that AQP9 is associated with 5-FU-based chemotherapy sensitivity in CRC. As a transmembrane channel, AQP9 first act as a gate to small-molecule drugs, which functionally controls 5-FU uptake and accumulation in cells; on the other hand, AQP9 may induce S-phase cell cycle arrest through activation of RAS and downstream signaling pathway, thus further increasing the cytotoxicity of S-phase-specific drug 5-FU that accumulated in CRC cells (Figure 7). That AQP9 is overexpressed in CRC chemotherapy responders raises the possibility that increasing the AQP9 level may be an efficient therapeutic approach in overcoming chemoresistance. Notably, AQP9 might be a biomarker that can predict the benefit of 5-FU-based chemotherapy, particularly in stage III CRC. Detection of AQP9 level before receiving chemotherapy may help CRC patients avoid invalid 5-FU-based treatment and select other effective regimens at early time.

## Materials and methods

### Reagents and vectors

For immunoblotting analysis, the following antibodies were used: anti-*β*-actin or anti-GAPDH as loading controls (Proteintech); anti-p-AKT (S473), anti-AKT, anti-p-GSK (Ser 9), anti-GSK3, anti-p-ERK (T202/Y204), anti-ERK, anti-p21, anti-Phospho-Histone H2A.X (Ser139), anti-cyclin D1 (Cell Signaling Technology, Danvers, MA, USA); anti-GFP (Proteintech, Chicago, IL, USA), and anti-AQP9 (Santa Cruz Biotech, Santa Cruz, CA, USA). For IHC, antibodies against AQP9 (Abcam, Cambridge, UK) were used. The pEGFP and pEGFP-AQP9 plasmid was obtained from Addgene (Cambridge, MA, USA). The pcDNA 3.1 (+) plasmid was purchased from Invitrogen (Carlsbad, CA, USA). 5-FU was purchased from Sigma (Cambridge, MA, USA).

### Patients and samples

For AQP9 mRNA quantification, 32 CRC samples (bulk samples) were obtained from patients who underwent surgery and were then treated with 5-FU based chemotherapy (mFOLFOX6 or XELOX) at the Sixth Affiliated Hospital of Sun Yat-sen University (SYSU) between 2009 and 2012. All specimens were immediately put in RNA*later* solution (Ambion, Carlsbad, CA, USA) after surgery and stored at −80 °C until RNA extraction. No patient received preoperative chemotherapy or radiotherapy. Patients who were deceased or had recurrent tumors within 2 years after chemotherapy were considered to be chemotherapy nonresponders, otherwise the patients were considered to be responders.

Tissue microarrays (TMA) were constructed using paraffin-embedded samples of primary colorectal adenocarcinomas patients at the Sixth Affiliated Hospital of SYSU from 2007 to 2012. Patient enrollment criteria included: pathological confirmation of CRC, the undergoing of curative surgical resection, absence of preoperative chemotherapy, availability of tumor specimen, and complete follow-up information. The median follow-up time was 1490 days (range 195–2636 days). OS or DFS was the end point of the study. OS time was calculated from the date of surgery to the date of death or the last follow-up time. DFS time was calculated from the date of surgery to the date of recurrence.

### TMA construction and IHC

The paraffin-embedded tissue blocks and the corresponding histological H&E-stained slides were overlaid for tissue TMA sampling. Duplicates of 1 mm diameter cylinders were punched from representative tumor areas of individual donor tissue block and re-embedded into a recipient paraffin block at a defined position, using a tissue-arraying instrument (Mini Core, Plaisir, France).

For IHC analysis, paraffin sections were incubated with primary antibody against AQP9 (1:1000). For negative control, isotype-matched antibodies were applied. To evaluate the AQP9 expression level, each slide was assigned a score for intensity and staining positive pattern. Immunostaining was evaluated independently by two pathologists. The percentage of positive tumor cells is as follows: 1 (up to 10% of positive cells), 2 (10–50% of positive cells), 3 (50–80% of positive cells) and 4 (>80% of positive cells). Intensity scores ranged from 0 to 3: 0, no staining; 1, weak; 2, moderate; and 3, strong. Multiplication of the two scores resulted in a final score ranging from 0 to 12. Receiver operation characteristic (ROC) curve analysis was applied to determine the cutoff point for tumor ‘high expression’ by using the 0, 1 criterion. Under these conditions, samples with scores <5 and scores ⩾5 were defined as low and high expression of AQP9, respectively.

### Cell culture and immunofluorescence

CRC cell lines were purchased from the American Type Culture Collection (ATCC, Manassas, VA, USA). HCT116 and DLD1 cells were maintained in RPMI-1640 (GIBCO), supplemented with 10% (v/v) fetal bovine serum (FBS, GIBCO, Carlsbad, CA, USA). Cells were allowed to grow in a humidified incubator with 5% CO_2_ at 37 °C. Mycoplasmas were detected in cell cultures stained with DAPI (Santa Cruz) as described elsewhere.^46^

Cells were fixed by 4% paraformaldehyde for 10 min at 4 °C. Fixed cells were then permeabilized in 0.5% Triton X-100 for 10 min and blocked with 5% BSA for 1 h. Primary antibody incubation was performed at 4 °C overnight. The cell nuclei were counterstained with DAPI. The images were analyzed using a SP8 (Leica, Wetzlar, Germany) confocal microscope.

### Plasmids construction and transfection

A series of human AQP9 mutants with NPA deletions, including ΔNPA1, ΔNPA2 and ΔNPA1+2, were generated by PCR mutagenesis. The fragments upstream and downstream of the NPA1 and NPA2 motifs were PCR-amplified and ligated to the vector, respectively. The plasmid was made by in-frame subcloning of human AQP9 cDNA into the pEGFP-C1 or pcDNA 3.1 (+) vector using the In-fusion HD Cloning Kit (Clonetech, Tokyo, Japan). All the plasmid constructs were confirmed by sequencing.

Cells cultured to 70% confluence were transfected with wild-type (WT) AQP9 and NPA mutants using Lipofectamine 3000 (Invitrogen). The transfectants, designated as HCT116_vec_, HCT116_AQP9-wt_ HCT116_AQP9-ΔNPA1_ HCT116_AQP9-ΔNPA2_ and HCT116_AQP9-ΔNPA1+2_, were selected with G418 at 0.8 mg/ml for stable transfection. Once selected, clones were tested by western blotting and maintained in RPMI-1640 medium containing 0.4 mg/ml G418.

### RNA preparation and real-time quantitative RT-PCR

Total RNAs from frozen tissue samples or CRC cell lines were extracted using TRIZOL reagent (Invitrogen) following the manufacturer’s protocol. A total of 1 *μ*g RNA from each sample was reverse transcribed using mixed oligo-dT/random primers and gDNA removal and the cDNA Synthesis Kit (TOYOBO, Tokyo, Japan). AQP9 mRNA levels were quantified by qPCR using the Applied Biosystems 7500 Real-Time PCR System (Carlsbad, CA, USA). The primers were: AQP9 Forward: 5′-CTTCCAGTTCCCGCTATGCTA-3′, Reverse: 5′-CTGAATGCCACAATGTCCTCC-3′ mRNA quantity was normalized using GAPDH as control (forward, 5′-GTCAACGGATTTGGTCTGTATT-3′ and reverse, 5′-AGTCTTCTGGGTGGCAGTGAT-3′) and fold change of expression was calculated according to the ^ΔΔ^CT method.

### Western blotting analysis

Total proteins were extracted from the cultured cells using RIPA Lysis and Extraction Buffer (Thermo scientific, Carlsbad, CA, USA). The protein concentration was equilibrated using the Bio-Rad DC Protein Assay Kit (Biorad, Hercules, CA, USA) and then subjected to 10% sodium dodecyl sulfate polyacrylamide gel electrophoresis (SDS-PAGE). The proteins separated on SDS-PAGE were transferred onto a PVDF membrane (Milipore, San Diego, CA, USA). The membrane was blocked for 1 h in Tris-Buffered saline and Tween 20 (TBST: 10 mM Tris-Cl, 150 mM NaCl and 0.05% Tween 20) containing 4% BSA. All primary antibodies were incubated overnight at 4 °C. HRP-linked anti-rabbit or mouse IgG secondary antibodies (Thermo scientific) were used to detect primary antibody binding. Protein was detected by ECL chemiluminescence system (Milipore) on autoradiography film (Kodak, Rochester, NY, USA) or ChemiDoc Touch (Bio-Rad).

### Real-time cell growth curve assay

For cells monitored by xCELLigence RTCA DP instrument (Roche, Basel, Switzerland), 10 000 cells per well were seeded on 16-well E-Plates (proliferation assays) from Roche. To test drug sensitivity, 5-FU were serially diluted with culture medium and applied to adhered cell cultures. Cell activities were expressed as the cell impedance index (CI) and were continuously monitored in a standard cell culture incubator at 37 °C and 5% CO_2_.

For cells monitored by IncuCyte ZOOM instrument (Essen BioScience, Ann Arbor, MA, USA), 10 000 cells per well were seeded on 96-well plates. Cells were placed in an IncuCyte ZOOM with a 10 × objective in a standard cell culture incubator at 37 °C and 5% CO_2_. Two images per well were collected every 2 h over 3 days in both phase-contrast and fluorescence. Specifically, images were segmented and cell confluence was calculated as cell surface area (mm^2^) for each time point using the IncuCyte software.

### Flow cytometric analysis of cell cycle and apoptosis

Flow cytometric analysis of HCT116 cell apoptosis was detected by using the Annexin V-FITC/Propidium Iodide (PI) Apoptosis Detection Kit (LiankeBio, Hangzhou, China). According to the manufacturer’s instruction, 1 × 10^6^ cells were washed twice in PBS before re-suspension in 50 *μ*l PBS with 2% FBS. Annexin V-FITC and PI of 5 *μ*l, respectively, were added and stained on ice for 30 min. The cells were re-suspended to 500 *μ*l PBS and flow cytometric (FACSII; BD Biosciences New York, NY, USA) analysis was conducted within 30 min. Cell apoptosis was detected by the Annexin V-PE/7-AAD Apoptosis Detection Kit for DLD1 cells transfected with GFP-AQP9.

To estimate the proportions of cells in various phases of the cell cycle, cellular DNA contents were also measured by flow cytometry. Cells transfected with AQP9 or control plasmids were trypsinized and then fixed in 75% ice-cold ethanol. The pellets were suspended in PBS and washed twice. The cells were stained with PI solution and then analyzed by flow cytometry. The rate of the cell cycle within G0/G1, S and G2/M phase was determined by analysis with the FLOWJOW 9.1 software (Tree Star, San Francisco, CA, USA).

### HPLC measurement of intracellular 5-FU concentration

The HPLC system used was Techcomp LC2000 (Techcomp, Beijing, China). The mobile phase consisted of methanol and water (5 : 95, v/v), filtered and degassed prior to use. The flow rate was 1 ml/min with UV detection at 266 nm.

For intracellular measurement of 5-FU concentration, CRC cell lines transfected with vector or AQP9 were incubated with 50 *μ*g/ml 5-FU at 37 °C for 24 h. Cells without 5-FU treatment were used as blank controls. Cells were washed with PBS for three times and suspended at a density of 10^6^ cells/600 *μ*l. Cell membranes were disrupted by ultrasonic at 80 Hz for 5 min over intervals. The supernatant was collected and cell debris was discarded. 5-FU standard solutions were prepared freshly prior to each experiment at final concentrations of 0, 20, 50, 100, 200, 500, 1000 and 2000 ng/ml. A standard curve was created using peak area against standard 5-FU concentrations. Prior to injection into the HPLC system, both cell samples and 5-FU standard solution were added with 1 ml ethyl acetate and then vortexed, dried and reconstituted in 120 *μ*l of mobile phase. The injection volume to HPLC system was 100 *μ*l. To determine the intracellular level of 5-FU, peak area was calculated for each experiment.

### Animal studies

Forty male BALB/c nude mice (6–8-week old) were obtained from Laboratory Animal Center of SYSU. Mice were randomly divided into four groups: group 1, mice injected with HCT116_AQP9_ cells and treated with PBS; group 2, mice injected with HCT116_vec_ cells and treated with PBS; group 3, mice injected with HCT116_AQP9_ cells and treated with 5-FU; and group 4, mice injected with HCT116_vec_ cells and treated with 5-FU. HCT116_AQP9_ or HCT116_vec_ cells suspended in 0.2 ml matrigel prepared with serum-free RPMI-1640 medium (volume 1:2; BD Biosciences) were used for tumor implantation. Approximately 5 × 10^6^ cells were subcutaneously injected into the right flanks of the mice. When tumor size reached >100 mm^3^ (2 weeks after injection), tumor-bearing animals were administrated with 5-FU or equal volume of sterilized PBS. 5-FU was dissolved in 100 *μ*l PBS and administered daily by injections (20 mg/kg/day) for 7 days. Tumor growth and mice body weight was observed and recorded over 2 weeks. Tumor volume was measured using digital calipers and was calculated using the formula: (length × width^2^)/2. At the end of the study, animals were killed by cervical dislocation and tumors were excised carefully for further analyses. The relative tumor volume for each mouse was calculated as Vn/V0, where Vn is the volume at a given time and V0 is the volume at the start of treatment. Results are expressed as the mean daily change in tumor volume for each group of mice.

Tumor tissue apoptosis was determined by TUNEL method using DeadEnd Flurometric TUNEL System (Promega, Madison, WI, USA) according to the manufacturer’s protocol. Briefly, paraffin sections were cleared in xylene for 5 min and rehydrated in decreasing concentration of ethanol, with incubation in 0.5% NaCl solution. After wash with PBS three times, DNA was linearized with an incubation of Proteinase K at room temperature for 20 min. Slides were then incubated in TUNEL reaction mixture at 37 °C for 1 h, protected from light. After three rinses with PBS, the samples were analyzed under a fluorescence microscope (Leica DMI 2000, Wetzlar, Germany) at excitation wavelength of 540 nm and emission wavelength of 580 nm. Slides were then counterstained with DAPI.

### Statistics

All of the experiments were repeated at least three times. Statistical analyses were performed using the GraphPad Prism 5 software (GraphPad, La Jolla, CA, USA). Statistical analyses for cell line experiments were performed by Student’s *t*-test or Mann–Whitney *U*-test. For *in vivo* assays in xenografts, statistical analyses were performed by two-way ANOVA. A *P*-value<0.05 was considered statistically significant (*P<0.05, **P<0.01, ***P<0.001).

OS was calculated from the date of surgery to the date of death or the last follow-up time if follow-up was >5 years. For DFS, an event was defined as the first clinical or pathological evidence of local or distant recurrence. ROC curve analysis was applied to determine the cutoff point for tumor ‘high expression’ by using the 0, 1 criterion. Under this condition, a score value of 5 was adopted as cutoff for stratification of AQP9 expression into low (<5, AQP9−) and high (⩾5, AQP9+).

The relationship between AQP9 and clinicopathological features of CRC patients was analyzed by chi-square test. The Kaplan–Meier method was used for the univariate survival analysis, and the differences between compared groups were assessed by the log-rank test. The Cox proportional hazards regression model was used to compare OS and DFS between marker categories and to obtain risk ratios.

### Study approval

A written informed consent from each patient regarding tissue sampling had been obtained and the study were reviewed and approved by the Medical Ethics Committee of the Sixth Affiliated Hospital, Sun Yat-sen University. The present studies in animals were reviewed and approved by the Animal Care and Use Committee of SYSU.

## Figures and Tables

**Table 1:** Correlation between expression of AQP9, chemotherapy status and clinicopathological features in 367 cases of colorectal cancer

**Factors**	***N***	**AQP9 expression**		***P-*****value**	**Chemotherapy**		***P-*****value**
		**Low**	**High**		**No**	**Yes**	
	367	198	169		133	234	
*Age, years*				0.917			<0.001
⩽60	182	99	83		35	147	
>60	185	99	86		98	87	
							
*Sex*				0.833			0.514
Male	207	113	94		72	135	
Female	160	85	75		61	99	
							
*pN status*				0.142			<0.001
N0	170	85	85		85	85	
N1–N3	197	113	84		48	149	
							
*Grade*				0.004			0.011
Well	105	44	61		49	56	
Low/moderate	262	154	108		84	178	
							
*Tumor location*				0.037			0.385
Rectum	189	112	77		64	125	
Colon	178	86	92		69	109	

**Table 2:** Multivariate analysis of disease-free survival and overall survival in 367 CRC patients

**Factors**	***N***	**DFS**		**OS**	
		**HR (95% CI)**	***P-*****value**	**HR (95% CI)**	***P-*****value**
*Age, years*		1.196 (0.792, 1.806)	0.395	1.366 (0.818, 2.283)	0.233
⩽60	182				
>60	185				
					
*Sex*		0.867 (0.589, 1.276)	0.469	0.999 (0.635, 1.571)	0.995
Male	207				
Female	160				
					
*pN status*		2.194 (1.419, 3.393)	<0.001	1.473 (0.950, 2.283)	<0.001
N0	171				
N1–N3	196				
					
*Grade*		1.439 (0.876, 2.362)	0.150	1.521 (0.831, 2.782)	0.174
Well	105				
Low/moderate	262				
					
*Tumor location*		0.795 (0.536, 1.177)	0.252	1.042 (0.658, 1.650)	0.862
Rectum	189				
Colon	178				
					
*Chemotherapy*		0.314 (0.156, 0.634)	0.001	0.148 (0.065, 0.340)	<0.001
No	133				
Yes	234				
					
*AQP9 expression*		0.680 (0.455, 1.108)	0.061	0.790 (0.492, 1.267)	0.327
Low	198				
High	169				

Abbreviations: CI, confidence interval; DFS, disease-free survival; HR, hazard ratio; OS, overall survival.
