# Supplementary material for: AQP9-induced cell cycle arrest is associated with RAS activation and improves chemotherapy treatment efficacy in colorectal cancer
Source: Cell Death Dis. 2017 Jun 22;8(6):e2894–. doi: 10.1038/cddis.2017.282 (PMC5520935; doi:10.1038/cddis.2017.282)
Supplement: Supplementary Tables [file cddis2017282x1.docx]

**Supplementary Table 1. Univariate and multivariate analysis of disease-free survival in 234 CRC patients with chemotherapy**

| **Factors** | **N** | | **Univariate analysis** | |  | **Multivariate analysis** | |
| --- | --- | --- | --- | --- | --- | --- | --- |
|  |  | **Mean DFS**  **(Months)** | | ***P*** |  | **HR**  **(95% CI)** | ***P*** |
| Age  <57  >=57 | 108  126 | 65.7  67.4 | | 0.878 |  | - | - |
| Sex  Male  Female | 135  99 | 63.6  71.8 | | 0.079 |  | - | - |
| pT status  1-2  3-4 | 14  220 | 70.3  67.1 | | 0.519 |  | - | - |
| pN status  N0  N1-N3 | 85  149 | 72.9  64.0 | | **0.044** |  | - | - |
| Grade  Well  Moderate | 53  181 | 72.6  65.3 | | 0.063 |  | - | - |
| Tumor location  Rectum  Colon | 125  109 | 61.8  73.2 | | **0.004** |  | 1.96 (1.15, 3.34) | **0.014** |
| AQP9 expression  Low  High | 134  100 | 62.7  73.3 | | **0.008** |  | 1.90 (1.09, 3.30) | **0.023** |

DFS: disease-free survival; CI: confidence interval; HR: hazard ratio

**Supplementary Table 2. Univariate and multivariate analysis of overall survival in 234 CRC patients with chemotherapy**

| **Factors** | **N** | | **Univariate analysis** | |  | **Multivariate analysis** | |
| --- | --- | --- | --- | --- | --- | --- | --- |
|  |  | **Mean OS**  **(Months)** | | ***P*** |  | **HR**  **(95% CI)** | ***P*** |
| Age  <57  >=57 | 108  126 | 77.7  75.2 | | 0.503 |  | - | - |
| Sex  Male  Female | 135  99 | 75.2  77.3 | | 0.669 |  | - | - |
| pT status  1-2  3-4 | 14  220 | 77.4  76.2 | | 0.671 |  | - | - |
| pN status  N0  N1-N3 | 85  149 | 81.7  72.9 | | **0.010** |  | 0.39 (0.17, 0.89) | **0.025** |
| Grade  Well  Moderate | 53  181 | 79.7  74.8 | | **0.019** |  | 0.26 (0.06, 1.09) | 0.067 |
| Tumor location  Rectum  Colon | 125  109 | 75.2  77.4 | | 0.271 |  | - | - |
| AQP9 expression  Low  High | 134  100 | 75.0  78.3 | | 0.301 |  | - | - |

OS: Overall survival; CI: confidence interval; HR: hazard ratio
