# Supplementary material for: AQP9-induced cell cycle arrest is associated with RAS activation and improves chemotherapy treatment efficacy in colorectal cancer
Source: Cell Death Dis. 2017 Jun 22;8(6):e2894–. doi: 10.1038/cddis.2017.282 (PMC5520935; doi:10.1038/cddis.2017.282)

Supplementary figure legends

Supplementary Figure 1. Correlation between expression of AQP9 and clinical outcome. (a) Tumor AQP9 expression was determined relative to GAPDH by qPCR. AQP9 expression in responders (n=16) was significantly higher than in nonresponders (n=16) (P = 0.013). (b) Correlation between AQP9 mRNA and primary tumor stage in CRC from the TCGA dataset. AQP9 mRNA quantity, as determined by RNA-Seq, was significantly higher stage pT3 than stage pT1-2 (P = 0.042). Unpaired *t*-test was performed to determine the statistical significance between groups. (c) Representative IHC staining images for AQP9 high expression and low expression in CRC tissue microarrays. Images were captured at magnifications of 100×; the inset shows a higher magnification of 400× (lower panel).

Supplementary Figure 2. Cell cycle analyses and immune blotting for corresponding markers in DLD1 cell line. (a) DLD1 cells transfected with control vector and AQP9, γH2AX (red) was analysed by immunofluorence. (b) HCT116 cells transfected with control vector and AQP9 were synchronized by serum starvation for 48h, and induced to re-enter the cell cycle by the addition of serum over 0 to 24h. Cells were harvested for PI staining and analyzed by FACS to determine the cell cycle fraction. (c) Activation of Ras signaling, p21, H2AX and GAPDH proteins were analyzed by Western blot analysis in DLD1 cells.

Supplementary Figure 3. Correlation between AQP9 level, glucose condition and KRAS status in HCT116 cells according to microarray data from BioGPS. High glucose (25mM) downregulates AQP9 mRNA level in KRAS-mutant CRC cells, but increases AQP9 level in wild-type KRAS CRC cells.

Supplementary Figure 1

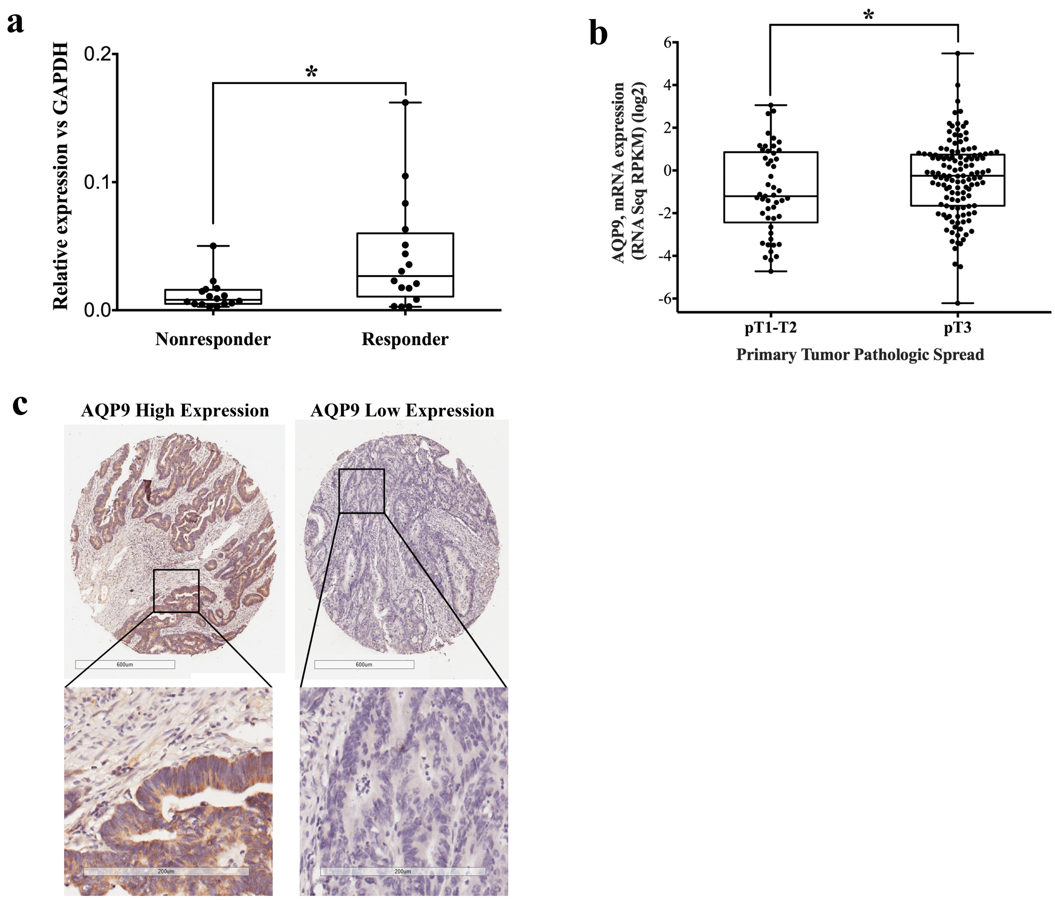

Supplementary Figure 2

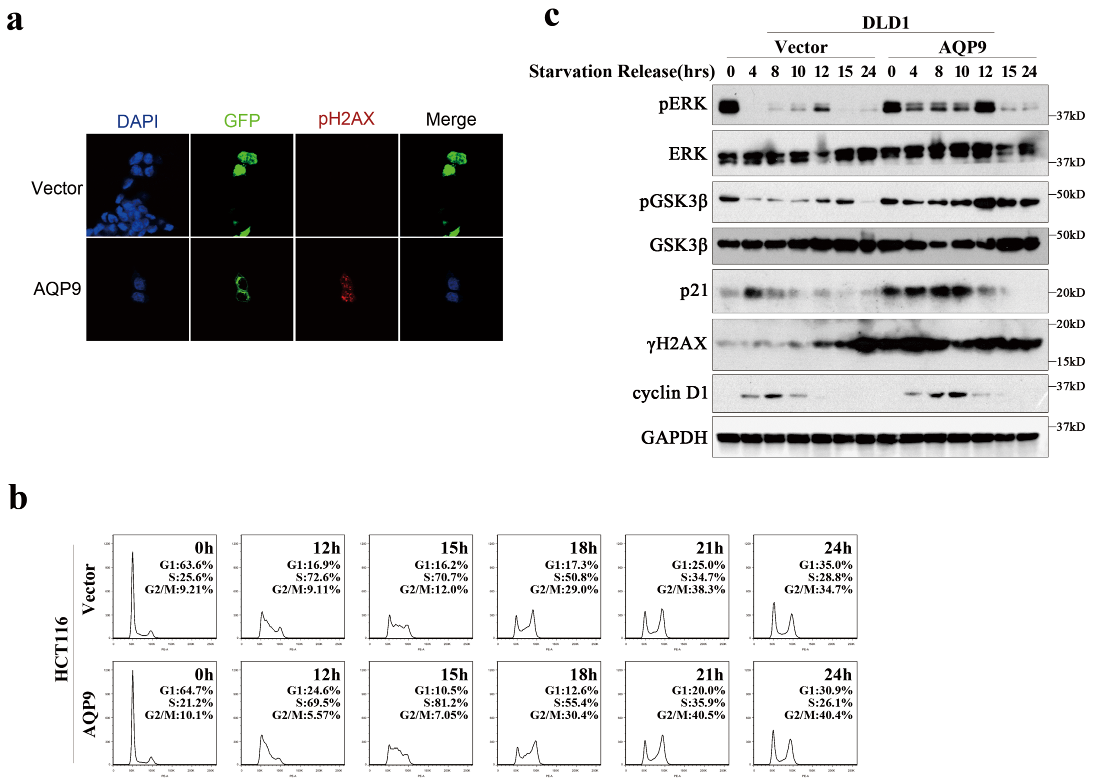

Supplementary Figure 3

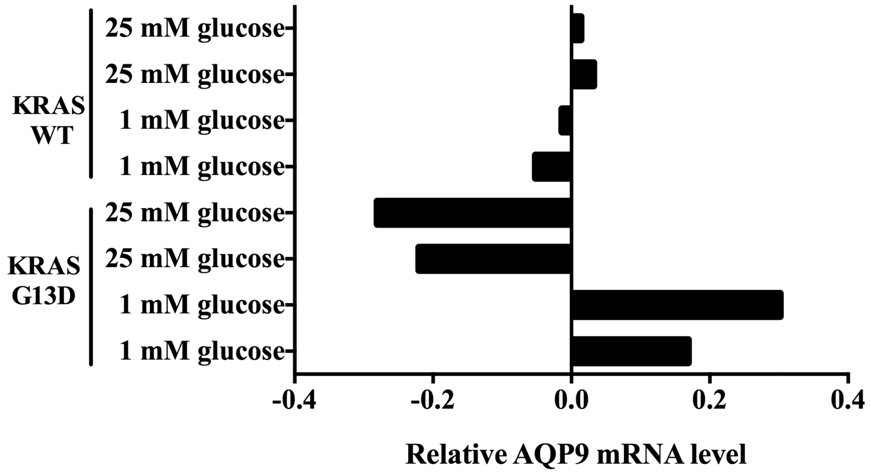

Supplement: Supplementary Figures and Legends [file cddis2017282x2.pdf]
